# Is MRI a viable alternative to CT/CBCT to identify the course of the inferior alveolar nerve in relation to the roots of the third molars?

**DOI:** 10.1007/s00784-020-03716-4

**Published:** 2020-12-07

**Authors:** Florian Beck, Stephanie Austermann, Kristina Bertl, Christian Ulm, Stefan Lettner, Andrea Toelly, André Gahleitner

**Affiliations:** 1 Division of Oral Surgery, University Clinic of Dentistry, Medical University of Vienna, Vienna, Austria; 2 Department of Periodontology, Faculty of Odontology, University of Malmö, Malmö, Sweden; 3 Karl Donath Laboratory for Hard Tissue and Biomaterial Research, Division of Oral Surgery, University Clinic of Dentistry, Medical University of Vienna, Vienna, Austria; 4 Department of Biomedical Imaging and Image-guided Therapy, Medical University of Vienna, Vienna, Austria

**Keywords:** Cone beam computed tomography, Magnetic resonance imaging, Multidetector computed tomography, Oral surgery, Third molar, Mandibular nerve

## Abstract

**Objectives:**

To assess the reliability of judging the spatial relation between the inferior alveolar nerve (IAN) and mandibular third molar (MTM) based on MRI or CT/CBCT images.

**Methods:**

Altogether, CT/CBCT and MRI images of 87 MTMs were examined twice by 3 examiners with different degrees of experience. The course of the IAN in relation to the MTM, the presence/absence of a direct contact between IAN and MTM, and the presence of accessory IAN were determined.

**Results:**

The IAN was in > 40% of the cases judged as inferior, while an interradicular position was diagnosed in < 5% of the cases. The overall agreement was good (*κ* = 0.72) and any disagreement between the imaging modalities was primarily among the adjacent regions, i.e., buccal/lingual/interradicular vs. inferior. CT/CBCT judgements presented a very good agreement for the inter- and intrarater comparison (*κ* > 0.80), while MRI judgements showed a slightly lower, but good agreement (*κ* = 0.74). A direct contact between IAN and MTM was diagnosed in about 65%, but in almost 20% a disagreement between the judgements based on MRI and CT/CBCT was present resulting in a moderate overall agreement (*κ* = 0.60). The agreement between the judgements based on MRI and CT/CBCT appeared independent of the examiner’s experience and accessory IAN were described in 10 cases in MRI compared to 3 cases in CT/CBCT images.

**Conclusions:**

A good inter- and intrarater agreement has been observed for the assessment of the spatial relation between the IAN and MTM based on MRI images. Further, MRI images might provide advantages in the detection of accessory IAN compared to CT/CBCT.

**Clinical relevance:**

MRI appears as viable alternative to CT/CBCT for preoperative assessment of the IAN in relation to the MTM.

**Supplementary Information:**

The online version contains supplementary material available at 10.1007/s00784-020-03716-4.

## Introduction

The inferior alveolar nerve (IAN) is next to the lingual nerve, one of the most endangered anatomical structures during the surgical removal of mandibular third molars (MTM). Any impairment (i.e., temporary or persistent) of the IAN after MTM removal is reported with an overall incidence of 3.6%; a persistent neurosensory deficit after 6 months is observed in 0.9% [1]. The probability of IAN impairment after MTM removal depends on the spatial relation between the mandibular canal (MC) and the MTM [2] and lack of a bony MC wall increases the risk of IAN impairment [3]. Hence, preoperative imaging is essential and should include at least a conventional radiograph [4]. In cases with a close relation between the MC and MTM, the exact distance and spatial relation might not be correctly assessed by 2D images making additional 3D imaging (i.e., computed tomography (CT) or cone beam computed tomography (CBCT)) necessary [4, 5]. However, CT/CBCT diagnostics relies on the presence of a bony MC wall for locating the IAN. A histomorphometric analysis indicated that the integrity of the MC wall is dependent on the quality/density of the surrounding trabecular bone [6], which explains that in approximately every fifth patient the IAN/MC appears difficult to be identified based on CBCT images [7].

In this context, magnetic resonance imaging (MRI) has been recently proposed as a radiation-free 3D imaging method for MTM [8]. MRI benefits by directly depicting the neurovascular bundle (NVB) [9] and is therefore independent of the integrity of the MC wall [10, 11]. An ex vivo study has proven by superimposition of MRI and CT scans that the geometric accuracy of MRI to display the IAN is comparable to the CT technique [12]. However, the shape and volume of the NVB were underestimated in a more recent study tracing the IAN in CBCT and MRI [13], and in 7% of the cases with 1 T MRI, the relation between the MC and impacted MTM was not assessable due to magnetic susceptibility artifacts [14]. Nevertheless, by using 3 T MRI, which has been demonstrated being superior for detection of the courses of brain nerves compared to 1.5 T MRI [15], the accurate visualization of the mandibular branch of the trigeminal nerve has been corroborated [16, 17].

The increasing acquisition and use of 3D radiographic imaging within dentistry (e.g., for implant treatment planning [18] and preoperative assessment of MTM [19, 20]) is raising the overall ionizing radiation exposure of the population, i.e., an additional increase of the cancer incidence of 0.46 per year was estimated due to applying CBCT imaging prior to MTM removal [21]. Therefore, MRI could be a reasonable alternative for the planning of various surgical procedures within dentistry [22]. However, despite the increasing number of studies reporting on the possibilities of IAN visualization by MRI, only a few studies [8, 14, 23] have addressed whether MRI is a reliable diagnostic method to evaluate the course of the IAN in relation to the roots of the MTM. These studies, which have used MRI images alone [14] or MRI with either panoramic radiographs [8] or CBCT [23], have only considered the vertical relation between the MTM and the MC on a panoramic overview. However, from a clinical point of view, the whole 3D volume including the bucco-lingual relation is important and might affect the surgical approach [24]. Thus, the aim of this study was to assess the reliability of judging the spatial relation between IAN/MC and the roots of the MTM based on orthoradial slices of MRI or CT/CBCT images.

## Material and Methods

### Study design and study population

This cross-sectional study was conducted at the Department of Radiology of the University Clinic of Dentistry, Medical University of Vienna (Austria) between 2017 and 2018. The protocol was approved by the local ethics committee (EK-Nr.: 1487/2017) and all patients provided written informed consent; reporting complies with the STROBE (“Strengthening the Reporting of Observational studies in Epidemiology”) guidelines (Appendix 1). Panoramic radiographs of patients consulting the dental clinic for surgical removal of MTM are routinely screened for a close relation between the IAN/MC and the MTM according to the criteria of Rood and Shehab [25] (i.e., darkening of the root, deflected roots, narrowing of the root, dark and bifid root, interruption of the white line(s), diversion of the MC, narrowing of the MC). All patients, which were based on these criteria selected for additional 3D imaging to clarify the spatial relation between the IAN/MC and MTM, were invited to participate, i.e., only patients deemed independent of the present study as in the need of 3D imaging were considered for inclusion. The following exclusion criteria were defined: (1) < 18 years; (2) installation of any metallic medical device (i.e., aneurysm clip, cochlea implant, defibrillator, insulin/patient-controlled analgesia pump, intrauterine device, intravascular stents/catheter, orthodontic appliance, orthopedic device, valvular transplant); (3) pregnancy; (4) anxiety/restlessness; (5) claustrophobia; (6) tattoos involving metallic particles; and (7) permanent piercings or ear-rings.

### Image acquisition—CT/CBCT

According to the clinic’s routine, which are following the “ALARA” (As Low As Reasonably Achievable) principle, either a CBCT or a CT was performed. Specifically, if both MTM were indicated for 3D imaging, a standard dental CT protocol was performed [26] (Somatom Sensation 4, Siemens Healthcare, Erlangen, Germany; 80 mAs, 120 kV, slice thickness: 0.5 mm, FOV: 100–120 mm, table feed: 1 mm, convolution kernel: U70u), while in cases of a single MTM, a CBCT scan was recorded (3D Accuitomo XYZ Slice View Tomograph, J. Morita Mfg. Corp., Kyoto, Japan; FOV: 40 mm, slice thickness: 0.25 mm). Axial slices from the CT scans were transformed to orthoradial multiplanar reconstructions (MPR) using a semi-automatic line drawn within the center of the jaw by 2 radiologic technologists, who have both > 20 years of experience. The horizontal plane on sagittal MPR was tilted according to Down’s mandibular plane (a tangent through the gonial angle and the lowest point of the symphysis).

### Image acquisition—MRI

**Fig. 1:**
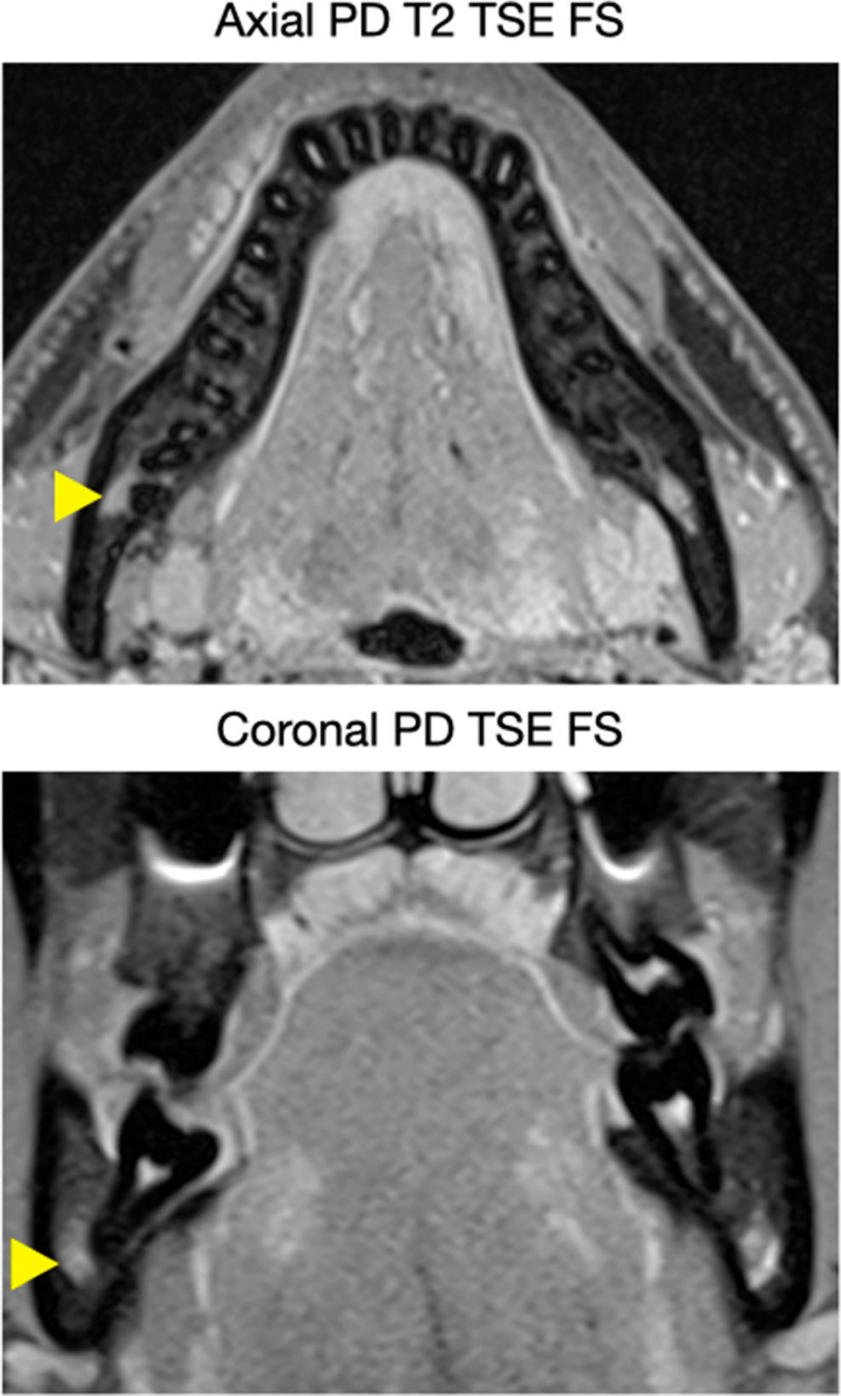
Two sequences (axial PD T2 TSE FS, coronal PD TSE FS) were selected for the identification of the IAN, which depicted teeth and cortical bone as hypointense, and the IAN (indicated by the yellow arrow), the pulp chamber, the periodontal ligament, small arteries, and dental follicles coronal to the MTM as hyperintense structures

MRI imaging was performed on a 3-T whole-body MRI scanner (MAGNETOM Skyra, Siemens Healthcare, Erlangen, Germany) using a 64-channel head/neck coil (Head/Neck 64 whole brain DSI, Siemens Healthcare, Erlangen, Germany). Two sequences (axial PD T2 TSE FS, coronal PD TSE FS) were selected by a radiologist (A.G.), specialized in musculoskeletal imaging, from the standard investigation protocol of the jaw region (Table 1, Fig. 1). All sequences were fat saturated to minimize the influence of fatty tissue from cancellous bone on IAN detection [27].

**Table 1:** MRI sequences and acquisition parameters.

	Axial PD T2 TSE	Coronal PD TSE
FOV	220 × 220 mm	154 × 170 mm
Matrix	320 × 320	320 × 232
Voxel size	0.34 × 0.34 × 2 mm^3^	0.27 × 0.27 × 2 mm^3^
TR	4060 ms	2780 ms
TE	9.5 ms	10 ms
Averages	1	3
Gap	2.6 mm	2.2 mm
Slice Thickness	2 mm	2 mm
Flip angle	138°	141°
Bandwidth	250 Hz/Px	300 Hz/Px
Acquisition time	4:15 (min:sec)	6:29 (min:sec)
FS	✓	✓
Acquisition type	2D	2D

*FOV* field of view, *FS* fat saturated, *PD* proton density, *TE* echo time, *TR* repetition time, *TSE* turbo spin echo

The images were imported in the OsiriX® DICOM image viewer (Pixmeo, SARL, Bernex, Switzerland) for further evaluation. Coronal PD TSE FS scans were transformed and tilted to orthoradial slices by using the “3D MPR” tool by a dental student (S.A.) under close supervision of an experienced radiologist (A.G.). For both imaging modalities, the orthoradial MPR have been produced prior to any assessment, i.e., all examiners judged the same reconstructions.

### Image evaluation and assessed parameters

**Fig. 2:**
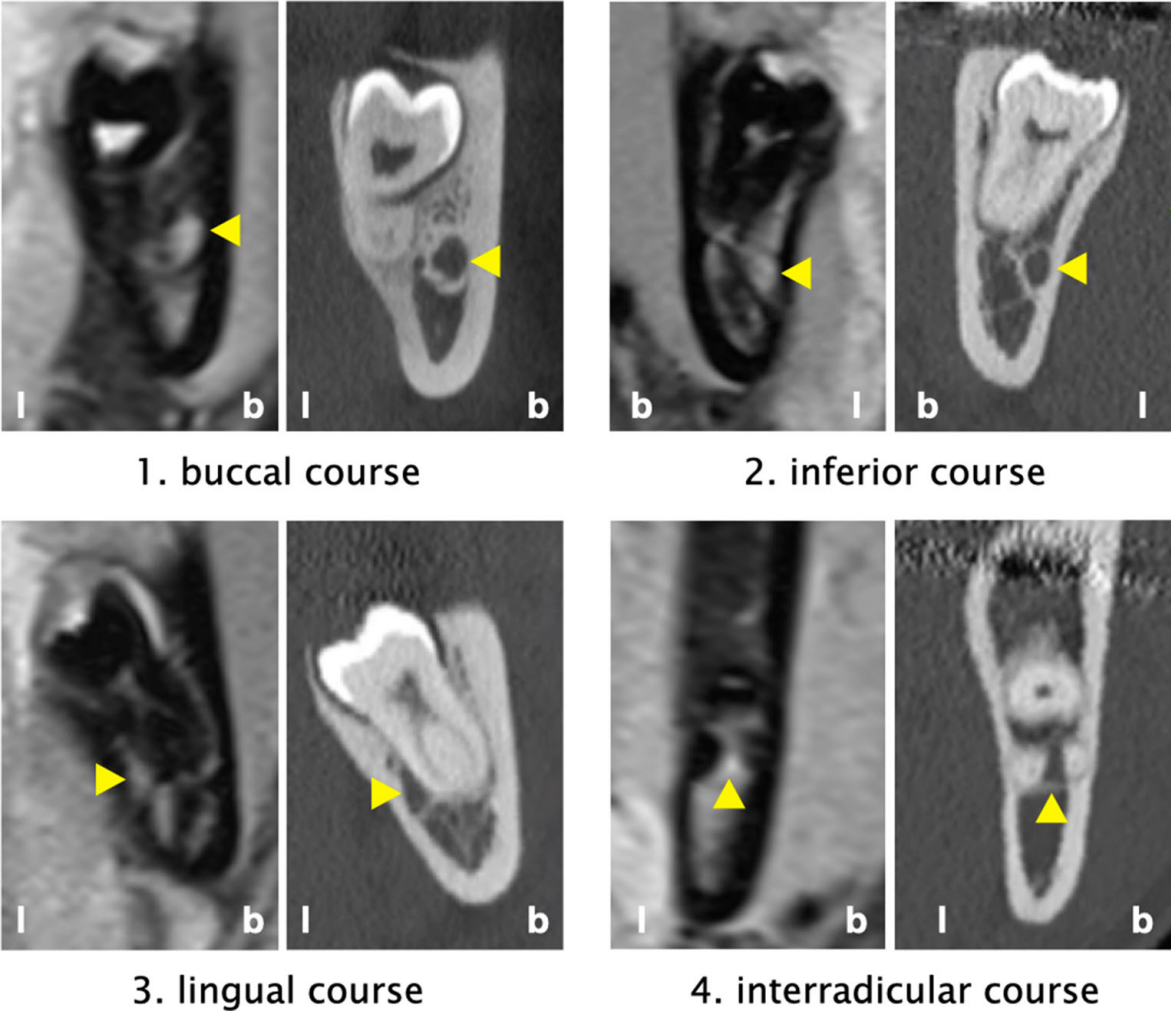
Classification of the course of the IAN/MC (indicated by the yellow arrow) as buccal, inferior, lingual, or interradicular. b, buccal aspect; l, lingual aspect

**Fig. 3:**
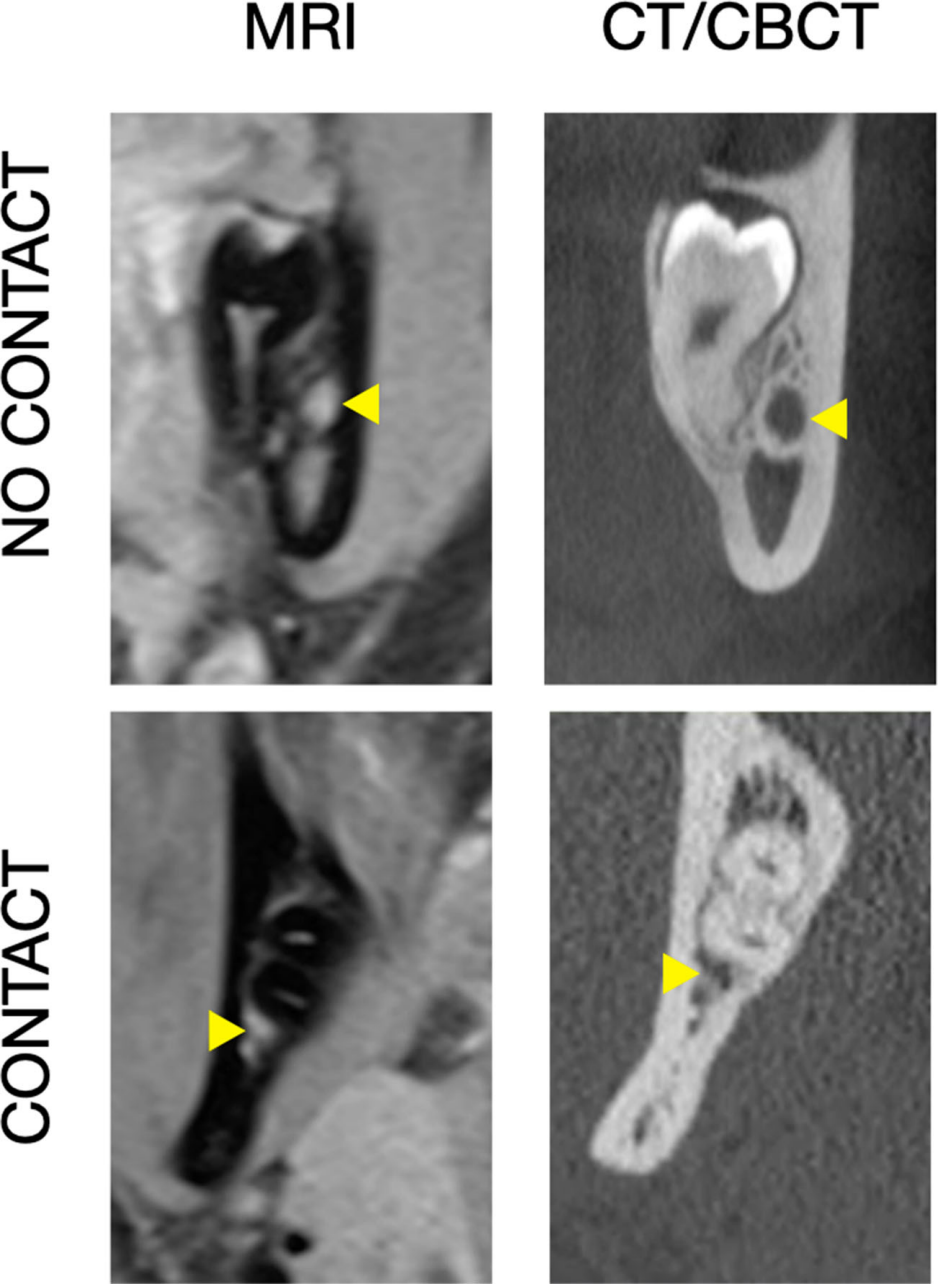
Presence/absence of bone tissue between the IAN/MC (indicated by the yellow arrow) and the roots of the MTM

**Fig. 4:**
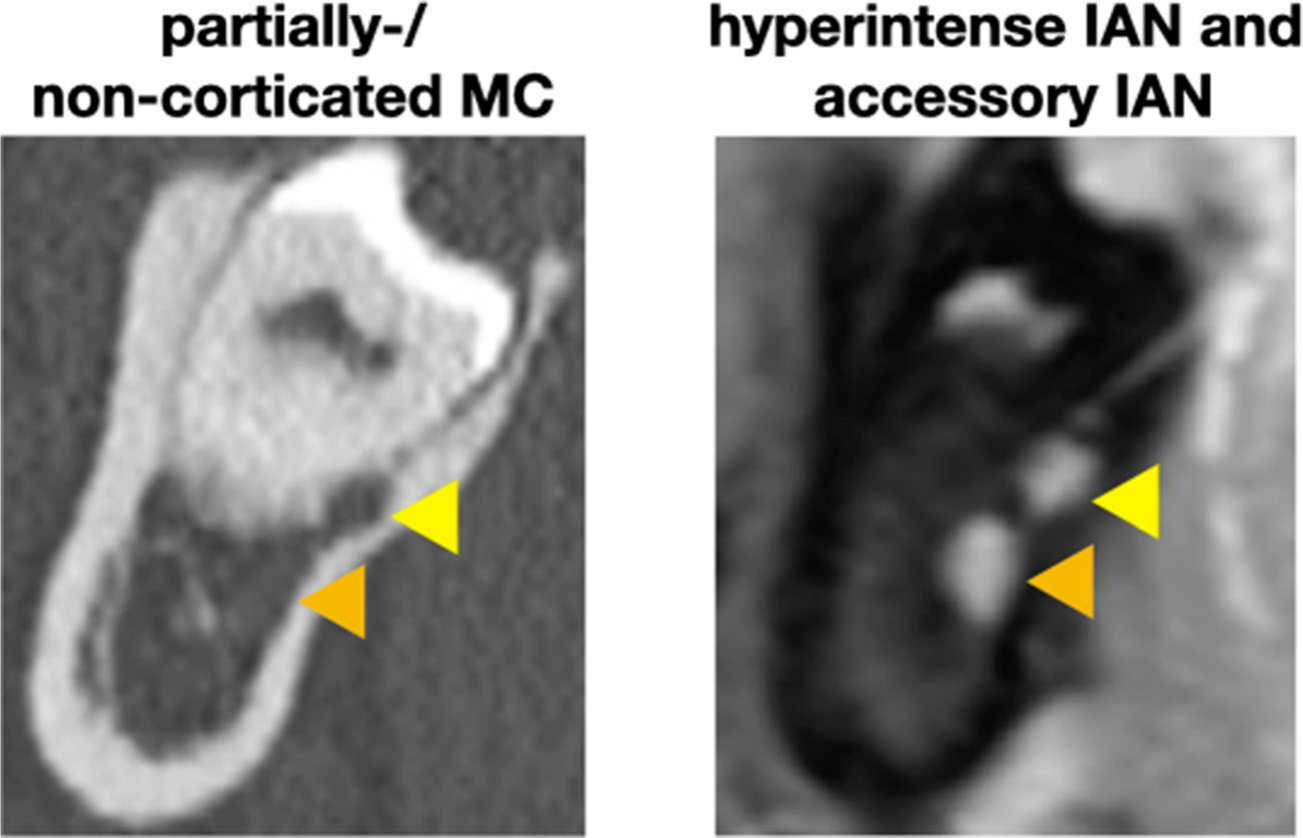
A case with the MC lacking a bony wall and with an accessory IAN. Left (CT): the MC (indicated by the orange arrow) is hardly visible and the accessory IAN (indicated by the yellow arrow) could be missed; right (MRI): both NVB appear clearly hyperintense

Image evaluation was performed independently on orthoradial MPR by 3 examiners with a different radiological background: (1) a radiologist (A.G.; > 15 years of experience), (2) an oral surgeon (F.B.; > 7 years of experience), and (3) a dental student (S.A.; last year of education). In a first step, all MRI scans were evaluated. Approximately 14 days later, all CT/CBCT scans were assessed, assuring that the evaluation of the MRI and CT/CBCT images of the same patient was performed independently and the raters had no possibility to change any previous judgement, i.e., each rating was immediately finalized and closed. The entire evaluation process was repeated by all 3 examiners after approximately 4 weeks. All evaluations were performed in the same room under standardized conditions on the same screen with the same settings. The following parameters were assessed on the MRI and CT/CBCT images:

- Course of the IAN/MC (primary outcome parameter): The course of the IAN/MC was classified according to Ghaeminia et al. [28] as (1) lingual, (2) interradicular, (3) buccal, or (4) inferior position (Fig. 2). The examiners were instructed to use for the classification the slice representing the closest position between the IAN/MC and the roots of the MTM. Courses matching 2 groups/classes (e.g., buccal and inferior) were allocated to the aspect, which harbored the greater cross-sectional area of the IAN; if cases were deemed as exactly matching 2 groups/classes (e.g., the cross-section was deemed as 50% buccal and 50% inferior), they were allocated to the aspect, which had a higher likelihood of IAN exposure during the surgical procedure.
- Presence/absence of a direct contact between the IAN and the roots of the MTM: Presence of a direct contact between the IAN and the roots of the MTM was defined as the absence of bone tissue between those 2 structures (Fig. 3).
- Presence of any accessory IAN/MC in the region of the MTM (Fig. 4)

In cases with a distal and mesial root, the course of the IAN/MC and the presence/absence of a direct contact were judged for each aspect separately; however, the exact root configuration was not part of the present study. Additionally, the following parameters were recorded: age, gender, and side of the investigated MTM. Prior to any assessment, the 3 examiners performed a calibration session based on 20 randomly chosen CT/CBCT and MRI images to discuss and evaluate the above-listed parameters and to dissolve any ambiguity.

### Sample size calculation

A sample size calculation was performed based on the likelihood of classifying the course of the IAN, the number of raters (*n* = 3), and an estimation of *κ*_E0_ = 0.6 from the literature [11, 16, 29]. A lower limit of *κ*_lower_ = 0.5 using the formula of Rotondi and Donner [30] was assumed in order to achieve a clinically relevant width with a 95% confidence interval (CI). The calculated sample size was 68 MTM.

### Statistical analysis

Fleiss’ kappa (*κ*) coefficients [31] were calculated for the judgements based on different imaging modalities (i.e., CT/CBCT vs. MRI) and for inter- and intrarater reliability of the 3 examiners for (1) the course of the IAN/MC and for (2) the presence/absence of a direct contact between the IAN and the roots of the MTM. The 95% CI of those coefficients were calculated by applying a hierarchical percentile bootstrap on the patient- and rater-level, in order to respect the structure of the data [32]. *P* values were computed by inverting those CI. The *κ* values were interpreted as (1) poor (< 0.2), (2) fair (0.21–0.4), (3) moderate (0.41–0.6), (4) good (0.61–0.8), and (5) very good (0.81–1.0) [33]. Further, the presence of an accessory IAN/MC in the region of the MTM was descriptively reported. All computations were done using R version 3.6.1 [34].

## Results

### Study population

The final study population consisted of 53 patients (33 female, 20 male; 29.5 ± 6.5 years) with 87 MTM, who completed both examinations (i.e., CT/CBCT and MRI) prior to MTM removal. Nineteen participants contributed with CBCT images of a single MTM, while in 34 participants, both MTM were examined by CT; the MTM distribution left to right was 39 to 48.

### MRI imaging technique

The MRI sequences (axial PD T2 TSE FS, coronal PD TSE FS), which were used for the identification of the IAN, depicted teeth and cortical bone as hypointense structures. The IAN, the pulp chamber (including the root canals), the periodontal ligament, small branches of arteries supplying the gingiva and teeth (Rami gingivales inferiores, Rami dentales inferiores), and dental follicles coronal to the MTM were displayed as hyperintense (Fig. 1). In none of the participants, metal artifacts were considered as having an impact on the judgement.

### Classification of the course of the IAN/MC

Table 2 presents the frequency distribution of the course of the IAN/MC in CT/CBCT and MRI based on all available judgements. For both imaging modalities the IAN/MC was most often judged as inferior (CT: 41.8%; MRI: 43.8%), followed by the buccal and lingual positions in about 24 to 30% of the cases, and an interradicular position of the IAN/MC was diagnosed by both imaging modalities in < 5% of the cases (CT: 3.7%; MRI: 3.1%). Further, Table 2 displays that a disagreement between the imaging modalities was primarily among the adjacent regions, i.e., primarily buccal/lingual vs. inferior and in some cases interradicular vs. inferior. Interestingly, in a few cases, the IAN/MC was judged as lingual in the CT/CBCT images, but as interradicular or buccal in the MRI images. The confusion buccal vs. lingual is based on 2 judgements of the dental student, while all 3 examiners judged the IAN/MC between 1 and 3 times in the MRI images as interradicular, although they judged it as lingual in the CT/CBCT images.

**Table 2:** Frequency distribution (%) of the course of the IAN/MC in CT/CBCT and MRI based on all available judgements

		Course of the IAN/MC in CT/CBCT				Total (MRI)
		Buccal	Inferior	Interradicular	Lingual	
Course of the IAN/MC in MRI	Buccal	21.5	7.2	0	0.2	28.9
	Inferior	2.5	33.9	1.1	6.3	43.8
	Interradicular	0	0	2.6	0.5	3.1
	lingual	0	0.7	0	23.5	24.2
Total (CT/CBCT)		24	41.8	3.7	30.5	100

*CT* computed tomography, *CBCT* cone beam computed tomography, *IAN* inferior alveolar nerve, *MC* mandibular canal, *MRI* magnetic resonance imaging

The overall agreement among all available judgements was good (*κ* = 0.72) with an absolute agreement in 81.5% of the cases. The CT/CBCT judgements presented in the inter- and intrarater comparison a very good agreement (interrater: *κ* = 0.81; intrarater: *κ* = 0.84), while the MRI judgements showed a slightly lower, but good agreement (interrater: *κ* = 0.74; intrarater: *κ* = 0.74). The maximum width of the corresponding 95% CI was 0.17 (i.e., the difference between *κ*_0.975_ and *κ*_0.025_; Table 3). A comparison of the *κ* values of the 3 examiners when assessing the agreement of their judgements CT/CBCT vs. MRI proved a good reliability independent of the experience (dental student: *κ* = 0.76; oral surgeon: *κ* = 0.76; radiologist: *κ* = 0.80) with an absolute agreement ranging from 74.1 to 86.5%.

**Table 3:** Kappa (κ) values for the course of the IAN/MC

		*κ*	*κ*_0.025_	*κ*_0.975_	*P* value
Overall		0.72	0.65	0.79	< 0.001
CT/CBCT	Interrater	0.81	0.76	0.86	< 0.001
	Intrarater	0.84	0.78	0.88	< 0.001
MRI	Interrater	0.74	0.67	0.80	< 0.001
	Intrarater	0.74	0.64	0.81	< 0.001

*CT* computed tomography, *CBCT* cone beam computed tomography, *MRI* magnetic resonance imaging

### Direct contact between the IAN/MC and the roots of the MTM

Table 4 presents the frequency distribution of a direct contact between the IAN/MC and the roots of the MTM in CT/CBCT and MRI based on all available judgements. For both imaging modalities, a direct contact between the IAN/MC and the roots of the MTM was diagnosed in about 65% (CT: 65%; MRI: 67.2%). However, in almost 20% of the judgements, a disagreement between CT/CBCT and MRI was detected.

**Table 4:** Frequency distribution (%) of a direct contact between the IAN/MC and the roots of the MTM in CT/CBCT and MRI based on all available judgements

		Contact CT/CBCT		Total (MRI)
		Yes	No	
Contact MRI	Yes	57.1	10.1	67.2
	No	7.9	24.9	32.8
Total (CT/CBCT)		65	35	100

*CT* computed tomography, *CBCT* cone beam computed tomography, *MRI* magnetic resonance imaging

The overall agreement among all available judgements was moderate (*κ* = 0.60) with an absolute agreement in 82% of the cases. Both image modalities presented a comparable, good inter- and intrarater agreement with the *κ* values ranging from 0.72 to 0.75. The maximum width of the corresponding 95% CI was 0.20 (i.e., the difference between *κ*_0.975_ and *κ*_0.025_; Table 5). A comparison of the *κ* values of the 3 examiners when assessing the agreement of their judgements CT/CBCT vs. MRI proved a moderate, close to good reliability independent of the experience (dental student: *κ* = 0.60; oral surgeon: *κ* = 0.59; radiologist: *κ* = 0.60) with an absolute agreement ranging from 80.2 to 83.0%.

**Table 5:** Kappa (*κ*) values of a direct contact between the IAN/MC and the roots of the MTM

		*κ*	*κ*_0.025_	*κ*_0.975_
Overall		0.60	0.49	0.69
CT/CBCT	Interrater	0.75	0.68	0.81
	Intrarater	0.72	0.63	0.79
MRI	Interrater	0.72	0.63	0.81
	Intrarater	0.74	0.65	0.83

*CT* computed tomography, *CBCT* cone beam computed tomography, *MRI* magnetic resonance imaging

### Presence of an accessory IAN/MC in the region of the MTM

The presence of an additional, separated hyperintense signal along the course of the IAN (i.e., an accessory IAN) was described in the region of 10 MTM of 10 different patients in the MRI images (Fig. 4). However, the raters did not agree in all cases, i.e., in 5 cases all examiners agreed on the presence of an accessory IAN, while in 2 cases 2 examiners and in 3 cases 1 examiner described an accessory IAN. The judgement of the CT/CBCT images revealed only in 3 cases an accessory MC, which was described in 2 cases by 2 examiners and in 1 case by only one examiner. Hence, in total, in 7 out of 87 cases (i.e., in 8.1% of the cases), an accessory IAN was assumed in the MRI images which was not reported in the CT/CBCT images (Fig. 4).

## Discussion

Taking the high frequency of MTM removal, the estimated increase of the cancer incidence due to applying CBCT imaging prior to MTM removal [21], and the questionable benefit to actually reduce the risk for neurosensory disturbance by CBCT compared to panoramic radiographs [35] into account, renders MRI as an interesting radiation-free 3D imaging method. The present study assessed the reliability of judging the spatial relation between IAN/MC and the roots of the MTM based on orthoradial slices of MRI or CT/CBCT images. A slightly lower inter- and intrarater agreement has been recorded for MRI compared to CT/CBCT images (i.e., *κ* of 0.74 vs. 0.81–0.84, respectively) when determining the course of the IAN/MC, but the recorded *κ* values still indicate a good reliability for the judgement of MRI images. Additionally, the agreement between the imaging modalities appeared independent of the examiner’s experience and MRI might offer advantages in selected cases with an accessory IAN. Herein, in about 8% of the cases, an accessory IAN was described in MRI images, which was not recorded in CT/CBCT images. The judgement of the direct contact between the IAN/MC and the roots of the MTM showed a higher disagreement between the imaging modalities (i.e., in almost 20% of the cases), which might be due to the fact that CT/CBCT and MRI is actually depicting the MC or the IAN, respectively.

**Fig. 5:**
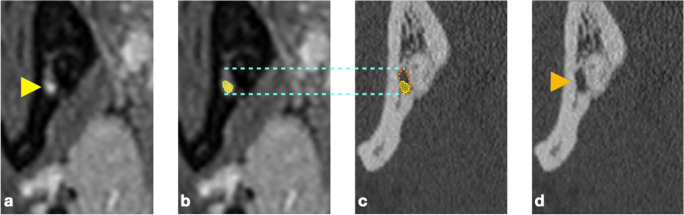
A case with the NVB (indicated in (**a**) by the yellow arrow and in (**b**, **c**) by the yellow area) not filling the whole volume of the MC (indicated in (**c**) by the orange dotted line and in (**d**) by the orange arrow); the corresponding vertical dimension of the MC between both imaging modalities (**b**, **c**) is indicated by the turquoise dotted line

In this context, CT/CBCT relies on the presence of a MC wall for locating the IAN, i.e., in cases with a minimal amount of bone surrounding the IAN, it can be difficult to identify the MC in CBCT images [7]. In contrast, MRI displays directly the NVB including the IAN. To date, there is no standard term regarding what is actually visualized by MRI. Nasel et al. [9] have previously stated that the neural and vascular structures within the MC could not be distinguished and thus referred to the term “NVB.” Since then, “NVB” [36–39], “IAN” [8, 17, 40, 41], “mandibular nerve” [10, 12], or “MC” [11, 14, 23, 29] were used synonymously in various publications. The cross-sectional area of the NVB in the region of the MTM measured by histomorphometry is about 13.45 ± 2.23 mm^2^; thereof, the IAN and the inferior alveolar artery (IAA) represent 32.4 and 4.5% of the area, respectively [39]. The IAN itself is composed of a larger mental (2/3) and a smaller incisive (1/3) branch [42]. In regard to the structural dimensions within the NVB (i.e., with the IAN being 6 to 7 times larger than the IAA), it is reasonable to assume that the hyperintense signal in MRI is mostly expressed by the IAN. However, the NVB as total does not necessarily fill the whole volume of the MC (Fig. 5). Additionally, the structures within the NVB might change their spatial relation to each other as it has been shown for the IAA, i.e., the IAA changes its position within the MC on average about 4 times being at the MTM most often in a cranial position [43]. Altogether, this might explain at least partly the higher disagreement observed herein between the imaging modalities for the judgement of a direct contact between the IAN/MC and the roots of the MTM. For example, the IAN might be located within the MC more distant to the roots and not displaying a direct contact in the MRI images, although no bone tissue is displayed between the MC and the roots in CT/CBCT images. Alternatively, the IAN might be located close to the roots and the hyperintense signal interferes with the judgement of the presence/absence of hypointense bone tissue between the IAN and the roots in MRI images. Finally, the superior visualization of hard tissue in CT/CBCT images (e.g., the border between cancellous bone and the root surface) might also play a role.

Any disagreement between the imaging modalities for the course of the IAN/MC was primarily among adjacent regions, i.e., primarily buccal/lingual vs. inferior and in some cases interradicular vs. inferior (Table 2). Specifically, the disagreement can be explained for the most part by the “borderline” cases with the IAN/MC matching 2 groups/classes (e.g., lingual and inferior). It was defined herein to allocate the IAN/MC to the aspect, which harbored the greater cross-sectional area; however, this implies naturally a certain risk for inter- and intrarater disagreement. Further, the fact that either the MC or the IAN are displayed in CT/CBCT or MRI images, respectively, probably contributes to a potential disagreement among adjacent groups/classes, i.e., as discussed above, that the IAN does not necessarily fill the whole volume of the MC and therefore might be depicted in a slightly different position in MRI images compared to CT/CBCT images. Nevertheless, only in very few cases (i.e., in 0.7% of all judgements) that the IAN/MC was clearly “misjudged” between the imaging modalities (i.e., a confusion between buccal/lingual, buccal/interradicular, or lingual/interradicular), which underlines MRI as viable alternative to CT/CBCT. Additionally, the confusion buccal vs. lingual appeared only twice by the examiner with the least experience. Finally, CT/CBCT was considered herein as a “gold standard” as it is the main 3D imaging technique being used if a close relation is suspected on panoramic radiographs [4]. However, one should keep in mind that the “misjudgment” might actually not necessarily occur when judging the MRI images. In fact, MRI is the most commonly used technique to directly visualize the IAN [22] and to precisely localize the IAN including pathologic processes [9, 44–46].

To the best of our knowledge, a direct comparison to previous studies is not possible, as this is the first comparison of MRI and CT/CBCT images for a description of the relation of IAN/MC to MTM based on a 3D volume data set. In general, this relation is ideally expressed by both the course of the IAN in relation to the roots of the MTM (i.e., buccal, lingual, inferior, interradicular) and the presence/absence of bone tissue between the IAN and the roots of the MTM [28]. For example, a previous study [8] compared amongst other parameters the vertical position of the MTM in relation to the IAN based on MRI and panoramic radiographs. They reached an agreement in only 56% of the cases and the interrater agreement was poor (*κ*_w_ = 0.142). However, from a clinical point of view, MRI will not replace the panoramic radiograph as primary diagnostic method, but—as shown herein—MRI could replace CT/CBCT in cases, where the panoramic radiograph indicated the need for an additional 3D imaging technique to display in detail the vertical and bucco-lingual relation between the IAN/MC and the roots of the MTM without additional ionizing radiation exposure.

Altogether, MRI presents several advantages compared to CT/CBCT. Besides the obvious ones, such as radiation-free recording and the independency of the presence of a bony MC wall, MRI might be advantageous in displaying an accessory IAN and/or the lingual nerve. Herein, in 7 out of 87 cases, an accessory IAN was described in the MRI images, which was not visible in the CT/CBCT images (Fig. 4). However, based on the present data, one cannot exclude the possibility of false positive results in the judgement of MRI images for an accessory IAN. Future studies including histological examinations would be required to determine the truth regarding the presence/absence of accessory IAN. In general, the actual lack of a “gold standard” should be considered when interpreting the results herein, i.e., one does actually not know whether the judgement based on MRI or CT/CBCT images is the truth. Further, a recent study explored the feasibility of MRI images to detect and follow the lingual nerve [47]. Visualization of the lingual nerve was not part of the present study and the best sequence to depict the lingual nerve requires future studies. In general, visualization of the lingual nerve prior to MTM removal sounds tempting, as a temporary neurosensory disturbance (up to 13 weeks) is reported in 2% of the cases [48] and the lingual nerve is not visible in CT/CBCT images. However, one should also keep in mind the limitations of MRI with the most important ones being the general accessibility and the fact that it is a more expensive and time-consuming examination. Specifically, the cost-effectiveness might be difficult to prove, especially as even CT/CBCT still lacks the proof of a benefit to actually reduce the risk for neurosensory disturbance compared to panoramic radiographs only [35].

When evaluating the results of the present study, several limitations should be taken into consideration. First, for both imaging modalities, the orthoradial MPR have been produced prior to any assessment by different persons. Specifically, 2 experienced radiologic technologists were responsible for the CT/CBCT scans and the dental student together with the radiologist for the MRI scans. It can be discussed whether orthoradial MPR performed by different persons may affect the reliability; however, as all examiners judged the same reconstructions, any effect should be minor. In this context, if the examiners would have prepared the MPR themselves, it might have lowered the reliability for both MRI and CT/CBCT. However, this might primarily affect the “borderline” cases with the IAN/MC matching 2 groups/classes. Second, the inclusion of a dental student could be considered as a weakness, yet it allowed us to get an idea about the effect of the examiner’s experience. Specifically, it was interesting to see that the agreement between the judgements of both imaging modalities appeared independent of the examiner’s experience for the outcome parameters (i.e., course of the IAN/MC and direct contact between the IAN/MC and the MTM). Hence, at least for this specific topic, already a short calibration session prior to committing this study enabled a dental student to read the MRI and CT/CBCT images with an agreement comparable to the 2 other examiners (i.e., an oral surgeon and a radiologist). This in turn allows a careful conclusion that one can probably expect a high learning curve when starting to incorporate MRI as an alternative to CT/CBCT for assessing the relation between IAN and MTM in daily practice. Third, 68 MTM were assessed herein by CT and only 19 cases by CBCT. As CBCT provides a slightly higher resolution compared to CT [49, 50], an assessment based on CBCT images only might increase the reliability of the “standard” method. Finally, the present study did neither aim to develop new sequences for head and neck MRI nor to compare different sequences. Instead, the investigation was based on sequences of the standard protocol, which are usually available in radiological institutions. Hence, it would be an interesting topic of future studies whether the depiction of the IAN could be further improved, which in turn might improve the reliability of judgements based on MRI images.

In conclusion, the present study demonstrated the suitability of MRI with fat-saturated axial PD T2 TSE and coronal PD TSE sequences for the assessment of the IAN course in relation to the roots of the MTM prior to surgery as a radiological method free of ionizing radiation. Specifically, although the judgement of MRI images showed a slightly lower inter- and intrarater agreement in terms of determining the course of the IAN/MC compared to a judgement based on CT/CBCT images, the reliability can be considered as good. Additionally, the agreement appeared overall independent of the examiner’s experience and MRI might have advantages in seldom cases with a second, accessory IAN.

## Supplementary Information

ESM 1 (DOC 79 kb)
